# Perioperative point-of-care-testing of plasmacholinesterases identifies older patients at risk for postoperative delirium: an observational prospective cohort study

**DOI:** 10.1186/s12877-023-04627-1

**Published:** 2024-02-06

**Authors:** Matthias S. Gruendel, Wibke Brenneisen, Jakob Wollborn, Gerrit Haaker, Melanie Meersch, Simone Gurlit, Ulrich Goebel

**Affiliations:** 1 Department of Anaesthesiology and Critical Care, St. Franziskus-Hospital GmbH, Hohenzollernring 70, Muenster, 48145 Germany; 2 Department of Anaesthesiology, Perioperative and Pain Medicine, Harvard Medical School, Brigham and Women’s Hospital, Boston, USA; 3 Department of Anaesthesiology, Intensive Care and Pain Medicine, University Hospital Muenster, Muenster, Germany; 4 Department of Public Health, District Council, Muenster, Germany

**Keywords:** Cholinesterases, Orthopaedics, Traumatology, Delirium

## Abstract

**Background:**

Postoperative delirium (POD) is a severe perioperative complication that may increase mortality and length-of-stay in older patients. Moreover, POD is a major economic burden to any healthcare system. An altered expression of Acetylcholine- and Butyrylcholinesterases (AChE, BuChE) due to an unbalanced neuroinflammatory response to trauma or an operative stimulus has been reported to play an essential role in the development of POD. We investigated if perioperative measurement of cholinesterases (ChEs) can help identifying patients at risk for the occurrence of POD in both, scheduled and emergency surgery patients.

**Methods:**

This monocentric prospective observational cohort study was performed in a tertiary hospital (departments of orthopaedic surgery and traumatology). One hundred and fifty-one patients aged above 75 years were enrolled for scheduled (*n* = 76) or trauma-related surgery (*n* = 75). Exclusion criteria were diagnosed dementia and anticholinergic medication. Plasma samples taken pre- and postoperatively were analysed regarding AChE and BuChE activity. Furthermore, perioperative assessment using different cognitive tests was performed. The type of anaesthesia (general vs. spinal anaesthesia) was analysed. Primary outcome was the incidence of POD assessed by the approved Confusion Assessment Method (CAM) in combination with the expression of AChE and BuChE.

**Results:**

Of 151 patients included, 38 (25.2%) suffered from POD; 11 (14%) in scheduled and 27 (36%) in emergency patients. AChE levels showed no difference throughout groups or time course.

Trauma patients had lower BuChE levels prior to surgery than scheduled patients (*p* < 0.001). Decline in BuChE levels correlated positively with the incidence of POD (1669 vs. 1175 U/l; *p* < 0.001). Emergency patients with BuChE levels below 1556 U/L were at highest risk for POD. There were no differences regarding length of stay between groups or incidence of POD. The type of anaesthesia had no influence regarding the incidence of POD. Only Charlson Comorbidity Index and Mini Nutrition Assessment demonstrated reliable strength in respect of POD.

**Conclusions:**

Perioperative measurement of BuChE activity can be used as a tool to identify patients at risk of POD. As a point-of-care test, quick results may alter the patients’ course prior to the development of POD.

**Trial registration:**

https://drks.de/search/de/trial/DRKS00017178.

## Background

Postoperative delirium (POD) is defined as an acute and varying disturbance in attention, cognition and awareness, evolving within a short time frame with a tendency of fluctuation, while not being related to any pre-existing neurological pathology, according to DSM-5 [1].

The incidence of postoperative delirium rises significantly depending on the age of patients and comorbidities, ranging from 10 up to 30% [2]. While delirium may develop in any individual following surgeries and interventions alike, emergency surgery shows 1.5 to 3-fold higher occurrence of delirum in older adults than on average [3]. Although physicians believe delirium is transient, systematic reviews have demonstrated that delirium can persist in up to 45% of patients after hospital discharge and in about 33% for one month after hospital discharge [4]. Risk factors for the persistence of delirium include age, dementia, relevant comorbidities (such as cardiac disease, diabetes or previous history of stroke), delirium severity and physical restraints [5]. Postoperative delirium is associated with a 10% higher risk of 30-day mortality and causes a relevant decline of clinical outcomes including longer ICU and hospital length of stay [6, 7]. Moreover, delirium is a major economic health-care burden, generating estimated additional costs of up to 24.000 USD per case [8].

There are numerous reasons for the development of delirium. Apart from pre-existing neurological dysfunction or dementia, anaemia, the application of drugs used for anaesthesia (such as benzodiazepines), hypoglycaemia, hypoxia, prolonged hypotension or significant changes in electrolyte concentration may trigger or increase POD [3]. Perioperative stress, altered cortisol levels, infections and changes in cytokine levels are hypothesized as contributing factors of POD [9]. The exact pathophysiological mechanism of delirium is still a subject of discussion, however there is evidence that neuroinflammatory processes and dysregulation of cholinergic neurotransmission play an important role in its development [10]. This neurotransmission is facilitated by the activation or suppression of cholinesterases (ChE), acetylcholinesterase (AChE) and butyrylcholinesterase (BuChE).

While expressed in almost any type of tissue, their biological function in the central nervous system (CNS) is still subject of ongoing research [11, 12]. In the brain, BuChE is mainly present in glia and white matter cells regulating cholinergic neurotransmission together with AChE but may also be present in specific neurons, mainly localized in the amygdala, hippocampus, and thalamus – regions of the brain responsible for emotions and short- and long-term memory [13, 14].

Prehospital as well as surgical trauma may induce an inflammatory stimulus which in turn is able to rapidly activate specific CD-68 positive microglia in the CNS [15]. This process of microglial activation is highly dependent on the inhibition of cholinergic transmission. Any alteration of acetylcholine levels (especially reduction in older patients) is referred to as a risk factor for POD. Plasma levels of AChE serve as a surrogate parameter for synaptic activity, while BuChE has a regulatory function via its AChE-hydrolyzing activity [16].

There has been an association of altered serum anticholinergic activity (SAA) as well as altered plasma activity of AChE and BuChE and development of POD [17]. This suggests that perioperative detection of those alterations – preferably before the development of POD – can be used to identify patients at risk and effectively adapt their course of treatment to prevent POD.

Therefore, the focus of this study was to evaluate the incidence of POD in both, scheduled- and trauma surgery and to identify the prognostic value of point-of-care analysis of AChE and BuChE activity.

## Methods

Institutional review board approval was obtained from the Research Ethics Committee of the Chamber of Physicians Westfalen–Lippe (https://www.aekwl.de/fuer-aerzte/ethik-kommission; IRB #2018–561-f-S) and written informed consent was obtained from all subjects participating in the trial prior to enrolment. The trial was registered prior to patient enrolment at https://drks.de (https://drks.de/search/de/trial/DRKS00017178, Principal investigator: Ulrich Goebel, Date of registration: April, 24th 2019) and followed the STROBE guidelines.

The study was conducted at the Department of Anaesthesiology and Critical Care and the departments of Orthopaedic Surgery and Traumatology at St. Franziskus Hospital Muenster (a tertiary care hospital), Germany. Inclusion criteria were age > 70 years and indication for scheduled or emergency surgery. Exclusion criteria were refusal to participate, diagnosed dementia or ongoing treatment with anticholinergic medication.

### Study design

After eligibility was confirmed, demographic data were collected and patients were immediately assessed for their individual POD risk using each of the following tests: Clock drawing test, Mini-mental status examination (MMSE), Frailty-Score (FS), Geriatric depression scale (GDS) and Mini-Nutrition Assessment (MNA) as well as Charlson comorbidity index (CCI) were obtained [18]. Additionally, duration of anaesthesia and surgery, type of anaesthetic procedure (general anaesthesia vs. spinal anaesthesia), need for ICU admission and ICU length of stay, overall length of hospital stay and physical status according to the American Society of Anesthesiologists (ASA PS) were recorded. Patients’ preoperative long-term medication was recorded with regards to a possible anticholinergic burden using the Anticholinergic drug scale (ADS).

The MMSE was performed prior to the operation in each group to ensure that the patients did not suffer from relevant limitations due to dementia, thus being eligible to a sufficient screening for delirium. For the scheduled surgery group, MMSE was assessed with the anaesthesiologic evaluation approx. two days prior to surgery. For emergency surgery, MMSE was performed directly prior to surgery, due to the need of immediate surgery.

Patients’ AChE- and BuChE activity were determined in a whole blood sample using a validated point-of-care test system (ELISA-CHE, Franz Köhler Chemie GmbH, Bensheim, Germany) following the manufacturer’s instructions. Measurements were performed within 24 h prior to surgery and on the first and third postoperative day.

Screening for the occurrence of POD was performed by a trained physician, blinded to the study design, using the Confusion Assessment Method (CAM). Patients were screened twice a day on the first, second and third postoperative day. The therapeutic regimen of patients participating in this study did not differ from regular treatment.

Detecting a delirium directly after the operation in the PACU seems difficult for a variety of reasons; one of them being the fact that post-anaesthesia hangover may influence an adequate detection of a delirium as measured by CAM. If a delirium was detected by the third postoperative day, the patient was marked as “delirium positive”. Treatment of the delirium positive patients was initiated after blood sampling of the third day, starting with a non-pharmacological approach according to our standards. Since we cannot exclude, that a pharmacological treatment may influence AChE or BuChE expression respectively, we chose to treat delirium after blood sampling on day three. Management of postoperative delirium included both, pharmacological and non-pharmacological treatment, according to the NICE guidelines [19].

### Outcome measures

The primary endpoint was the incidence of POD. Secondary endpoints included the difference in pre- and postoperative AChE-/BuChE activity between patients developing POD vs. patients without POD and correlation between preoperative risk factors, incidence of POD and AChE-/BuChE activity in patients with POD.

### Statistical analysis

Sample size calculation was performed using GPower (V 3.1.9.7) and R (V 3.5.1) for the primary hypothesis (*p*-value 0.05, Power > 80%) and a minimum of 150 patients—distributed equally between the two cohorts—were required. For every metric item, mean and standard deviation (SD) or median and minimum, maximum and range values were calculated and for categorical data frequencies and percentages were given. The Kolmogorov–Smirnov test was used to analyse variable distribution before statistical testing. First, univariate analyses were performed. Student’s t-test was used for normally distributed data and Mann Whitney U test for non-normally distributed data. Categorical variables were analysed with *X*^*2*^ test. Cholinesterase activity change over time in each group was assessed using repeated measures ANOVA, differences between groups were assessed using univariate ANOVA.

Binomial logistic regression analysis was performed to investigate the association between cholinesterase activity (for each time point separately) and POD and other confounding factors showing odds ratios and 95% confidence intervals (CI).

Continuous variables were dichotomized according to the 25% percentile (AChE and BuChE). Only variables showing statistically significant differences were included in further analysis. The initially unadjusted model was fit, followed by a multivariable model adjusting for the following variables: Age > 75, Diabetes, ASA > 2 and following items for risk stratification of POD: CDT, CCI, MNA, MMSE, GDS and FS.

A *p*-value of ≤ 0.05 was considered statistically significant. To adjust for multiple testing, a Bonferroni correction was subsequently performed, where necessary. All analyzes were performed using SPSS Statistics (Version 26, 2020 by SPSS Inc., Chicago, Illinois, USA).

## Results

Between May 2019 and August 2021, 246 patients were screened for eligibility. Ninety-five patients were excluded due to missing the inclusion criteria (*n* = 75), declining to participate (*n* = 11) or other reasons (*n* = 9). Finally, 151 patients were included in this trial. Of these, 76 patients underwent scheduled surgery, while 75 patients underwent emergency surgery due to trauma; all of these were included in the final analysis. There were no dropouts or any kind of retraction of the study´s approval of any patient or any missing data throughout the study population and study period.

Demographics showed that most of the patients in this study were female (*n* = 106, 70.2%). ASA physical status was evenly split between ASA 1 and 2 as well as ASA 3 and 4. 80% of patients suffered from hypertension, followed by congestive heart failure, coronary heart disease and chronic kidney failure (24%, 22% and 17% respectively), previous stroke or a history of diabetes (9% and 21% respectively) and hypercholesterolemia (21%). While scheduled surgery included hip and knee surgery, emergency procedures were hip and arm surgery, mainly (all Table 1).

**Table 1:** Demographics of all patients

Characteristic	*n* = 151, (100%)
Age, mean (SD), years	81,4 (6,6)
Female, No. (%)	106 (70,2)
Male, No. (%)	45 (29,8)
BMI, mean (SD), kg/m^2^	26,43 (4,97)
ASA Score	
1 or 2	78 (51,7%)
3 or 4	73 (48,3%)
**Relevant Comorbidities, No. (%)**	
Hypertension	121 (80,1)
Congestive heart failure	37 (24,5)
Coronary heart disease	33 (21,9)
Chronic kidney failure	26 (17,2)
Stroke	14 (9,3)
Diabetes	32 (21,2)
Hypothyreoidism	22 (14,6)
Hypercholesteremia	21 (13,9)
**Scheduled procedures; No. (%)**	76 (50,4)
Elective hip replacement	54 (35,8)
Elective knee replacement	22 (14,6)
**Emergency procedures, No. (%)**	75 (49,6)
Hip replacement	69 (45,7)
Humeral osteosynthesis	6 (3,9)
**Anesthesia specification**	
Anesthesia time (min)	122 (101–145)
Surgical duration time (min)	62 (48–80)
Length of hospital stay (days)	11 (9–13)
Spinal anesthesia (n)	103 (68,2%)
General anesthesia (n)	48 (31,8%)
Admission to ICU (n)	11 (7,3%)
Length of stay ICU (days)	2 (1–7)

*Abbreviation: n* Number of patients, data are given as mean and standard deviation or as median and interquartile range or percentage

Anaesthetic procedures included spinal anaesthesia for hip or knee surgery in both groups or general anaesthesia if chosen by the patient or medically necessary. General anesthesia was performed as a balanced anaesthesia with propofol, sufentanil, rocuronium and sevoflurane. Neuromuscular blocking state was monitored using the train-of-four and double-burst method at the end of surgery and prior to extubation. No patient received any antagonizing drugs such as neostigmine or sugammadex. The depth of general anaesthesia was monitored using bispectral index. The mean duration of anaesthesia was 122 min (range 92–148 min.) for general anaesthesia, while spinal anaesthesia vanished over time. The mean duration of surgical procedures was 62 min (range 48–80 min.). Blood loss, fluid management and the use of vasopressors was comparable in both groups.

All patients received opioid treatment for postoperative pain control. Only 11 patients were admitted to the ICU postoperatively. Table 1 gives a detailed overview regarding these items.

Of 151 patients included, 38 (25.2%) developed POD as assessed by CAM testing on postoperative days 1, 2 and 3. Preoperative risk stratification with different tests revealed significant differences between groups (Table 2). Charlson Comorbidity Index was significantly higher in POD patients (*p* < 0.001) and clock drawing was associated with a significantly lower score in patients with POD (*p* < 0.001). Preoperative Frailty Score was significantly higher in POD patients (*p* < 0.001) and nutrition assessment revealed the risk of malnutrition in POD patients (*p* < 0.001), all Table 2.

**Table 2:** Comparison POD vs. no-POD

**Characteristic**	**Total** ***n* = 151, (100%)**	**POD** ***n* = 38, (25,2%)**	**No-POD** ***n* = 113, (74,8%)**	***P*-value**
Age, mean (SD), years	81.4 (6.6)	83.2 (7.5)	80.8 (6.2)	0.090^a^
Female, No. (%)	106 (70,2%)	24 (63,2%)	82 (72,6%)	0,308^b^
Male, No. (%)	45 (29,8%)	14 36,8%)	31 (27,4%)	
BMI, mean (SD), kg/m^2^	26,43 ± 4,97	25,22 ± 4,28	26,84 ± 5,14	0,051^a^
ASA Score				
1 or 2	78 (51,7%)	15 (39,5%)	63 (55,7%)	0,222^b^
3 or 4	73 (48,3%)	23 (60,5)	50 (44,3%)	
Anticholineric drug scale	1 (1–3)	1 (2–3)	1 (0–2)	0,702^a^
Pre-operative MMSE	28,2 ± 1,9 (26–30)	28,3 ± 1,8 (24–30)	28,1 ± 2,1 (26–30)	0.711^a^
Charlson comorbidities index	4 (4–5)	5 (4–7)	4 (3–5)	** < 0,001**^**a**^
Clock drawing test	7 (5–10)	5 (0–7)	10 (7–10)	** < 0,001**^**a**^
Frailty score	2 (1–3)	3 (2–3)	1 (1–2)	** < 0,001**^**a**^
Mini Nutrition Assessment	12 (12–13)	12 (10–12)	13 (12–13)	** < 0,001**^**a**^
Length of anesthesia (min)	122 (101–145)	108 (92–148)	122 (104–144)	0,201^a^
Length of operation (min)	62 (48–80)	62 (44–84)	62 (50–79)	0,630^a^
Length of hospital stay (days)	11 (9–13)	11 (8–14)	11 (9–13)	0,737^a^
Type of anesthesia				
Spinal anesthesia	103 (68,2%)	22 (57,9%)	81 (71,7%)	0,158^b^
General anesthesia	48 (31,8%)	16 (42,1%)	32 (28,3%)	
Admission to ICU	11 (7,3%)	4 (10,5%)	7 (6,2%)	0,376^a^
Length of ICU stay (days)	2 (1–7)	3 (1–19)	2 (1–7)	0,494^a^

Demographics are given as mean and standard deviation, other data and tests are given as median and interquartile range or percentage unless otherwise indicated

*Abbreviation: n* Number of individuals

^a^Mann-Whitney-U

^b^Chi-Square test

Overall, MMSE value in all patients was 28,2 ± 1,9 (POD patients overall = 28,3 ± 1,8 and No-POD patients overall = 28,1 ± 2) showing no differences between the groups (*p* = 0.711, Table 2).

**Fig. 1:**
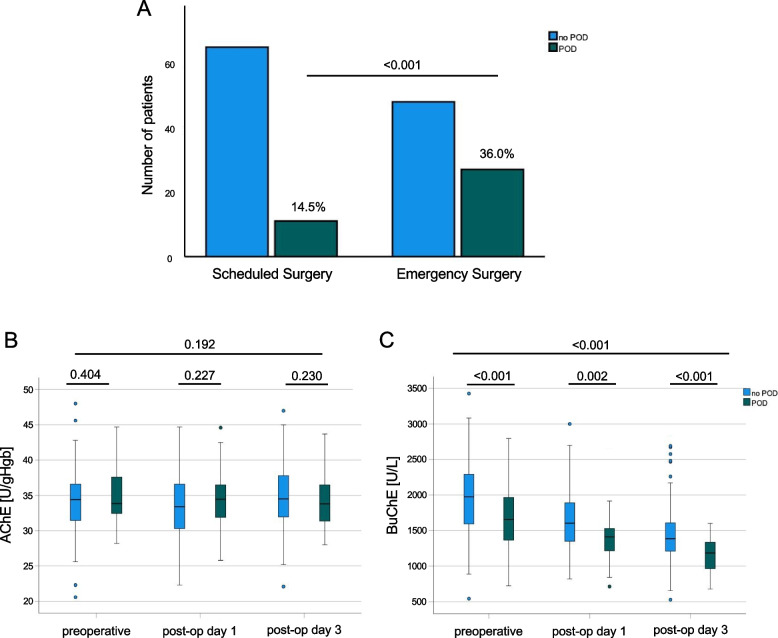
**A** Overall incidence of patients with POD in scheduled and emergency surgery. Data are total numbers; *p*-values are given exact unless lower than 0.001. **B** Serum parameter AChE [U/gHb] in all patients with and without POD preoperatively, on postoperative day 1 and on postoperative day 3; data are shown as mean and upper and lower quartile and whiskers; *p*-values are given exact. **C** Serum parameter BuChE [U/L] in all patients with and without POD preoperatively, on postoperative day 1 and on postoperative day 3; data are shown as mean and upper and lower quartile and whiskers; *p*-values are given exact unless lower than 0.001

In scheduled surgery 11 patients suffered from POD while 27 patients developed POD following emergency surgery (14.5% vs. 36%, *p* < 0.001) (Fig. 1A).

POD positive patients undergoing emergency surgery were significantly older than those who did not suffer from POD (*p* = 0.048) while there was no difference in scheduled surgery. Moreover, female sex was more pronounced regarding POD in emergency surgery than in the comparable groups (Table 3).

**Table 3:** Univariate analysis of the type of surgery and the expression of POD or no-POD

**Characteristic**	**Scheduled Surgery**		**Emergency Surgery**		***P*-value within group**		***P*-value between groups**
	**POD** ***n* = 11, (14,5%)**	**No-POD** ***n* = 65, (85,5%)**	**POD** ***n* = 27, (36,0%)**	**No-POD** ***n* = 48, (64,0%)**	**Scheduled**	**Emergency**	**overall**
Age (years)	80,8 ± 4,4 (77–89)	78,1 ± 4,5 (75–87)	86,4 ± 7,5 (75–99)	83,4 ± 6,6 (76–99)	0.070^a^	**0.048**^**a**^	0.090^a^
Female, No. (%)	9 (81,8%)	40 (61,5%)	19 (70,4%)	38 (79,2%)	0.972^b^	**0.026**^**b**^	0.308^b^
Male, No. (%)	2 (18,2%)	25 (38,5%)	8 (29,6%)	10 (20,8%)			
BMI, mean (SD), kg/m^2^	24,50 ± 4,33	27,29 ± 5,23	25,52 ± 4,31	26,25 ± 5,01	0.098^a^	0.282^a^	0.051^a^
ASA							
1 or 2	3 (27,3%)	44 (67,7%)	12 (44,4%)	19 (39,6%)	**0.035**^**b**^	0.082^b^	0.222^b^
3 or 4	8 (72,7%)	21 (32,3%)	15 (55,6%)	29 (60,4%)			
Anticholineric Drug Scale	1 (0–1)	0 (0–1)	1 (0–2)	1 (0–2)	0.225^a^	0.417^a^	0.702^a^
Pre-operative MMSE	27,4 ± 3 (25–30)	28,4 ± 1,5 (27–30)	28,0 ± 2,3 (24–30)	28,4 ± 1,5 (26–30)	0.081^a^	0.453^a^	0.195^a^
Charlson Comorbidities Index	4 (3–6)	4 (3–5)	6 (4–8)	4 (4–5)	0.195^a^	**0.017**^**a**^	** < 0.001**^**a**^
Clock drawing test	9 (7–10)	10 (7–10)	0 (0–5)	7 (5–10)	0.387^a^	** < 0.001**^**a**^	** < 0.001**^**a**^
Frailty score	2 (1–3)	1 (0–2)	3 (2–3)	2 (1–2)	**0.037**^**a**^	** < 0.001**^**a**^	** < 0.001**^**a**^
Mini Nutrition Assessment	12 (12–13)	13 (12–14)	11 (10–12)	12 (11–13)	0.131^a^	** < 0.001**^**a**^	** < 0.001**^**a**^
Length of anesthesia (min)	97 (79–149)	123 (108–139)	125 (97–148)	121 (102–153)	0.144^a^	0.497^a^	0.201^a^
Length of operation (min)	46 (40–85)	60 (51–78)	63 (45–83)	65 (47–93)	0.215^a^	0.93^a^	0.630^a^
Length of hospital stay (days)	11 (9–14)	11 (10–12)	10 (8–14)	10 (8–15)	0.594^a^	0.786^a^	0.737^a^
Type of anesthesia							
Spinal anesthesia	5 (45,5%)	54 (83,1%)	16 (59,3%)	27 56,3%)	**0.032**^**b**^	0.800^b^	0.158^b^
General anesthesia	6 (54,5%)	11 (16,9%)	11 (40,7%)	21 (43,8%)			
Admission to ICU	1 (9,1%)	0	3 (11,1%)	7 (14,6%)	n.v	0.673^a^	0.376^a^
Length of ICU stay (days)	1 (1–1)	0	3 (2–3)	2 (1–7)	n.v	0.199^a^	0.494^a^

Demographics are given as mean and standard deviation, other data and tests are given as median and interquartile range or percentage unless otherwise indicated

*n* Number of individuals, *n.v.* No value

^a^Mann–Whitney-U

^b^Chi-Square test

To guarantee for adequate screening regarding preoperative dementia in both groups, the MMSE analysis was performed. Subgroup analysis of the patients in the “scheduled surgery” group revealed MMSE values in POD patients (27,4 ± 3) and no-POD patients (28,4 ± 1,5); *p* = 0.081. The subgroup analysis of the patients in the “emergency surgery” group revealed MMSE values in POD patients (28,0 ± 2,3) and no-POD patients (28,4 ± 1,5); *p* = 0.453 (all Table 3). Values above 26 points account for no measurable dementia.

In addition, we calculated the multivariate binary logistic regression for the MMSE in combination with the expression of BuChE. The OR is 0.955, while the 95% CI is 0.816, 1.315 with a *p*-value of 0.428 (Table 5).

Preoperative scoring instruments all revealed significance in POD positive patients in emergency surgery, while only the Frailty score was associated with a significant result regarding POD in scheduled surgery (*p* = 0.037). Of note, patients undergoing scheduled surgery mainly received spinal anaesthesia procedures (83%). Among those receiving general anaesthesia in scheduled surgery, patients suffered significantly more often from POD (*p* = 0.032). In contrast, anaesthetic management had no influence on the incidence of POD in emergency surgery or the overall calculation (Table 3).

Length of anaesthesia, length of operative procedure, admission to or length of stay in the ICU as well as the overall length of hospital stay showed no difference within or between groups.

### Measurements of AChE and BuChE

**Fig. 2:**
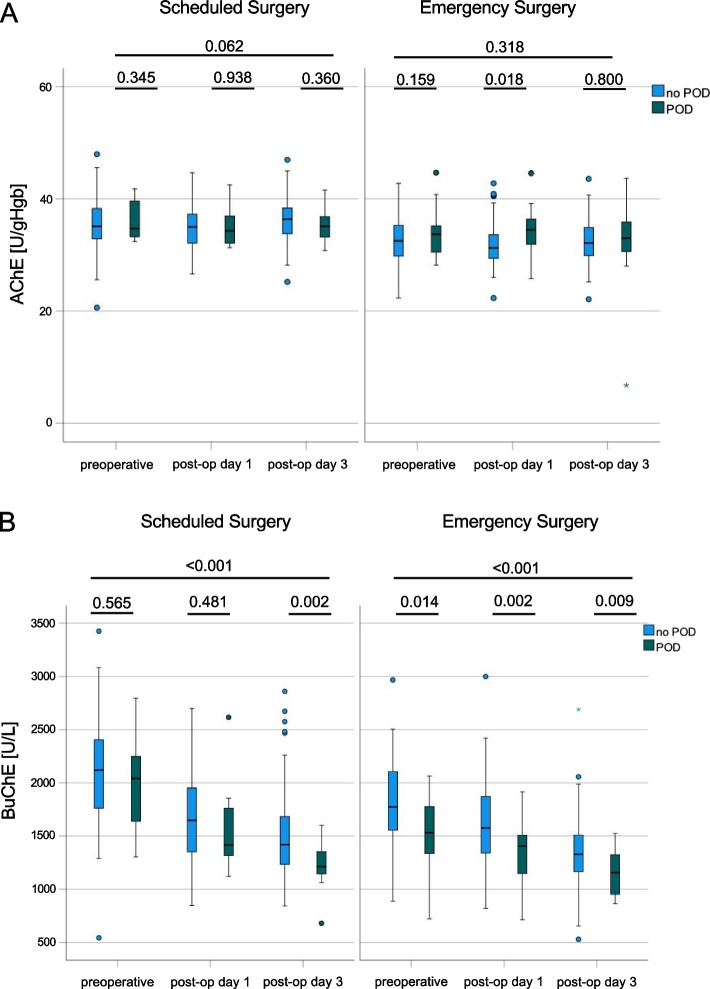
**A** Serum parameters AChE [U/gHb] in scheduled vs. emergency surgery in patients with or without POD preoperatively, on postoperative day 1 and on postoperative day 3; data are shown as mean and upper and lower quartile and whiskers; *p*-values are given exact unless lower than 0.001. **B** Serum parameters BuChE [U/L] in scheduled vs. emergency surgery in patients with or without POD preoperatively, on postoperative day 1 and on postoperative day 3; data are shown as mean and upper and lower quartile and whiskers; *p*-values are given exact unless lower than 0.001

AChE did not depict any differences, neither between preoperative and postoperative measurements on day 1 or 3 after surgery within the group (POD: day 1 vs. day 3: 34.88 vs. 33.6 U/gHb; *p* = 0.192 and No-POD: day 1 vs. day 3: 34.17 vs. 34.73 U/gHb; *p* = 0.37), nor between patients suffering from POD or those who did not at any given timepoint (all *p* > 0.05). Furthermore, there were no differences between the emergency or scheduled surgery groups concerning AChE in POD or no-POD patients overall (Table 4). AChE values were comparable at the preoperative time point (Fig. 1B). In the differentiated analysis of either scheduled or emergency surgery, a significant difference occurred on the first postoperative day between groups (*p* = 0.018, Fig. 2A).

**Table 4:** AChE and BuChE values in POD and No-POD patients in both groups overall

**Variable**	**Timepoint**	**POD** ***n* = 38, (25,2%)**		**No-POD** ***n* = 113, (74,8%)**		**ANOVA**	
		M	SD	M	SD	Groups	Time
						*p*-value	*p*-value
AChE	pre-operation	34,88	4,35	34,17	4,6	0.404	POD = 0.192
	postoperative day 1	34,78	3,89	33,82	4,35	0.227	No-POD = 0.37
	postoperative day 3	33,6	5,89	34,73	4,56	0.230	
BuChE	pre-operation	1669,5	470,85	1958,7	479,71	** < 0.001**	POD < **0.001**
	postoperative day 1	1406,98	340,43	1647,46	409,82	**0.002**	No-POD < **0.001**
	postoperative day 3	1175,01	220,15	1449,66	420,66	** < 0.001**	

*Abbreviations: n* Number of individuals, *m* Mean, *SD* Standard deviation

In contrast, BuChE values between groups differed significantly prior to surgery. Patients suffering from POD had significantly lower BuChE values before surgery (1669 ± 470 vs. 1958 ± 479 units/l, *p* < 0.001), which was even more pronounced in the following postoperative measurements (1406 ± 340 vs. 1647 ± 409 units, *p* = 0.002 on postoperative day 1 and 1175 ± 220 vs 1449 ± 420 units/l, *p* < 0.001 on postoperative day 3). There was a significant decline in BuChE regarding both groups (with and without POD) over time course (*p* < 0.001, all Table 4).

The more detailed analysis of BuChE expression confirmed these results: in scheduled surgery, there was a significant difference on postoperative day 3 between POD and no-POD patients (1218 ± 237 vs. 1523 ± 435 units, *p* = 0.002) and the overall decline over time was significant (*p* < 0.001; Fig. 1C). Emergency surgery patients suffering from POD showed significantly lower BuChE values prior to surgery (1537 ± 411 vs. 1788 ± 417 units, *p* = 0.014), with this trend continuing on the postoperative days (day 1: 1333 ± 281 vs. 1597 ± 413 units, *p* = 0.002 and day 3: 1155 ± 214 vs. 1345 ± 379 units, *p* = 0.009; Fig. 2B).

Using the preoperative expression of BuChE, we determined the 25th percentile as a cutoff value for increased risk of POD. A result of 1556 U/L was calculated, thus giving a valuable criterium for risk stratification regarding the development of POD *after* scheduled and emergency surgery. Patients in scheduled surgery that expressed BuChE levels below this threshold showed an increased risk of 17% of developing POD, while patients below this threshold in emergency surgery had an increased risk of 33% compared with the entire cohort.

To further analyse for confounding variables contributing to this decline in BuChE activity, we chose three variables to control in this context; age above 75 years, history of diabetes and ASA > 2 [20]. Additionally, the aforementioned preoperative tests for risk stratification were also analysed.

### Preoperative POD testing

Multivariate binary logistic regression for independent risk factor analysis revealed an OR of 1.433 ([95% CI, 1.014–2.024], *p* = 0.041) for the Charlson Comorbidity Index and OR 0.831 [95% CI, 0.579–1.194], *p* = 0.045) for Mini-Nutrition Assessment. The other preoperative tests for risk stratification, especially the Clock Drawing Test and Frailty Score, as well as age and history of diabetes did not appear to be significant confounding variables (Table 5).

**Table 5:** Multivariable binary logistic regression analysis for BuChE as an independent risk factor

Study parameter	OR	95% CI	*P*-value
Age > 75	2.279	0.427, 12.167	0.335
Diabetes	0.901	0.752, 1.078	0.069
ASA > 2	1.239	0.401, 3.832	0.709
Mini Mental Status Exam	0.955	0.816, 1.315	0.428
Clock Drawing Test	0.901	0.752, 1.078	0.254
CCI	1.433	1.014, 2.024	**0.041**
MNA	0.636	0.409, 0.990	**0.045**
GDS	0.831	0.579, 1.194	0.317
FS	1.814	0.869, 3.786	0.112

Data are given as odds ratio with 95% confidence interval

## Discussion

This study aimed to evaluate the incidence of POD in scheduled patients compared to trauma-related emergency surgery and the association of ChEs in this context. The main results of this single-center investigation may be summarized as follows: among elderly patients undergoing orthopedic procedures, the incidence of POD was significantly higher in emergency surgeries than in scheduled surgeries, while the type of anesthesia had no influence on POD. AChE activity did not differ significantly between groups or time points.

While BuChE activity showed an overall decline in all patients, it was significantly lower in patients developing POD, both between cohorts (scheduled vs. emergency surgery) and between pre- and postoperative measurements.

BuChE levels below the cutoff value of 1556 U/l showed an increased risk for later development of POD in both cohorts; therefore, it may be a useful tool for preoperative risk stratification independent of other confounding variables such as age or history of diabetes. The Charlson Comorbidity Index and Mini Nutrition Assessment showed the most promising reliability of the other tests used for the preoperative risk assessment of POD in elderly patients.

Postoperative delirium occurs within the first three days after surgery [21]. Data regarding the incidence of POD vary considerably, ranging from 3% up to 50% in high-risk patients [21–23]. Within this substantial range, emergency procedures are thought to present the strongest risk factor, with a three-fold higher risk compared to scheduled surgery [21]. Our data suggest an overall incidence of POD of 25%, with 14% in scheduled and 36% in emergency surgery. This data is comparable to recently published studies of mixed cohorts [24, 25].

While the type of anaesthesia is still discussed controversially, the overall incidence of POD in this trial did not differ between general anaesthesia or spinal anaesthesia only. Ravi et al. found that general anaesthesia was an independent risk factor for the development of POD [26]. Our results indicate a significantly lower incidence of POD in patients with spinal anaesthesia, but only for scheduled surgery. In contrast, Neuman et al. could not confirm these results, concluding that spinal anaesthesia was not superior to general anaesthesia in hip fracture surgery [27].

The plasma activity of AChE and BuChE may be altered due to a variety of stimuli including inflammation, major trauma, burns and sepsis [28–30]. Inhibition of AChE is currently under investigation in Alzheimer’s disease and vascular dementia, emphasizing the importance of ChE in neurological diseases [31].

Recent studies emphasized a mechanistic role of both, AChE and BuChE with varying conclusions in critically ill patients as well as in cardiac and non-cardiac surgery and even extracorporeal circulation [24, 32, 33].

In this study, AChE activity did not show any time course alteration or association with POD, in agreement with the data of Michels et al. [25] While the CESARO trial found significantly higher AChE activity in POD patients, we were not able to confirm this data [24].

Regarding BuChE, the current literature seems more consistent. Both, the CESARO trial and the secondary analysis of CESARO demonstrated a continuous decline in BuChE activity over time course, being associated with the occurrence of POD [24, 25]. In this study, preoperative BuChE activity was significantly lower in patients developing POD regardless of the surgical group. A significant decline in BuChE activity could be detected in all patients; those developing POD showed significantly lower BuChE activity throughout the postoperative period.

In line with current hypotheses regarding the pathophysiology of POD [9] this suggests that cholinergic moderation of neuroinflammation seems to be altered in POD patients. Following this hypothesis, we specifically chose to compare patients undergoing emergency and scheduled surgery to establish if recent trauma contributes to a decrease in ChE activity thus making development of POD more likely.

The expression of BuChE may also be analysed in cerebrospinal fluid (CSF). Lin et al. found that BuChE activity showed the most accurate diagnostic value concerning the later onset of POD compared to AChE, choline acetyltransferase and different interleukins [34].

In the current study, we tried to identify a cut-off value to get a prognostic score. We chose the 25% percentile using POD patients of both groups. With a cut-off value of 1556 U/L BuChE, a possibility of one-in-three was calculated to positively identify a patient at risk for POD. Of note, Zivkovic et al. estimated a critical BuChE level of 1661 U/L with a 94% sensitivity to best predict patient outcome in sepsis [30]. Although a different setting, BuChE cut-off values are almost identical.

In contrast to these findings, we suggest that low initial activity of BuChE or a substantial decrease of BuChE activity may represent a key factor in POD. Rump et al. postulate, that a midazolam-induced overexpression of BuChE may contribute to POD via an increased hydrolysis of acetylcholine [35]. These data are conflicting with the current POSE data, showing no increase in POD due to the use of midazolam [36].

John et al. found no association between AChE / BuChE expression and POD in their trial of cardiac surgical patients using extracorporeal circulation [32].

Among the many tests used to receive a precise summary of patients, all tests showed high significance in univariate testing condition. With multivariate testing, only Charlson Comorbidity Index and Mini Nutrition Assessment demonstrated reliable strength. This shows, while being time-consuming and labour-intensive, many tests fail to reliably identify patients at risk for POD.

The MMSE revealed that there was no significant difference within or between groups regarding POD. While our patients in the emergency group may be considered as “borderline” in respect to the diagnosis of dementia, this may be attributed to age and situation of any accident leading to hospital admission and prompt surgery. Overall, the patients in this study did not suffer from dementia, which may be a confounding factor in the expression of ChE.

The quantification of plasmacholinesterases represents a quick and easy, cheap and reliable procedure to identify older patients at risk of POD, which may easily be included in an everyday work routine. As a direct consequence, patients with low BuChE levels may be treated either by a specialized team using non-pharmacological methods or receive individual medication to counteract a potential POD (or both) as soon as these specific considerations arise.

### Limitations

This study was designed to evaluate the incidence of postoperative delirium both in elderly patients undergoing emergency surgery and scheduled surgery. Due to the nature of the study design, there was no possibility of randomization. Each investigator had a distinct part, blinded against the other results and assuring unbiased testing, such as measurement of ChE and the screening for POD using CAM twice a day postoperatively. Bedside point-of-care analysis minimizes the probability of error due to handling, freezing, and working samples for several days.

This study has some limitations. Patient observation was limited to the third postoperative day, without any further follow-up. The number of patients included was calculated for the incidence of postoperative delirium and assessment of ChE values. For all the other reported values, the number of patients was too low.

We did not measure the severity of POD (such as CAM-S) or the duration of the delirium until resolving respectively. Therefore, we cannot exclude any dose–response relation between pre-operative ChE activities and POD. Furthermore, we did not differentiate the type of POD (hypo- vs. hyperactive or mixed forms) which may have an influence on the expression of ChE.

The MMST was used to evaluate dementia prior to ChE testing. While pre-diagnosed dementia was an exclusion criterium, our cohort may be limited concerning the normal distribution of this diagnosis. This is reflected by the similar MMST scores in emergency vs. scheduled surgery groups.

Another limitation of this study is the mono-centric setting that may represent a bias regarding data interpretation.

The gender ratio has shifted in favour of female individuals, which clearly represents the overall demographics. However, male individuals could show different cut-off values for BuChE, thus altering our results in an evenly distributed population.

Due to the low number of patients, it was not possible to compare the predictive value of different tests for risk stratification regarding the likelihood of POD. In larger populations, the predictive value of an individual test or a combination of tests may be higher.

## Conclusion

Patients undergoing emergency surgery have a higher risk for POD than in scheduled surgery. Preoperative values and the decline of BuChE activity appear to be valuable and reliable progression parameters in the due course of older patients, while it is tempting to speculate about its preoperative prognostic quality to identify patients at risk for POD. Further studies in larger, more distinct cohorts like patients suffering from sepsis are necessary and should include patients with anticholinergic medication since these are more and more common among older patients.

## Data Availability

The datasets used and/or analysed during the current study are available from the corresponding author on reasonable request.

## Abbreviations

ASA PS: American Society of Anesthesiology Physical Status
AChE: Acetylcholinesterase
ADS: Anticholinergic Drug Scale
BuChE: Butyrylcholinesterase
CAM: Confusion Assessment Method
CCI: Charlson Comorbidity Index
CI: Confidence interval
ChE: Cholinesterase(s)
CNS: Central nervous system
CSF: Cerebrospinal fluid
DSM-5: Diagnostic and Statistical Manual of Mental Disorders, Version 5
FS: Frailty Score
Hb: Haemoglobin
GDS: Geriatric Depression Scale
ICU: Intensive Care Unit
M: Mean
min: Minute
MMSE: Mini-Mental Status Examination
MNA: Mini-Nutrition Assessment
no-POD: No postoperative delirium
OR: Odds ratio
p: *P*-value
POD: Postoperative delirium
SD: Standard deviation
